# Nitrogen donor ligand for capping ZnS quantum dots: a quantum chemical and toxicological insight

**DOI:** 10.1039/c9ra05651c

**Published:** 2019-09-11

**Authors:** Vivek Pandey, Vinay Kumar Tripathi, Keshav Kumar Singh, Tejasvi Bhatia, Nitesh Kumar Upadhyay, Bela Goyal, Gajanan Pandey, Inho Hwang, Poonam Tandon

**Affiliations:** Department of Physics, University of Lucknow Lucknow-226007 India; Department of Animal Science and Biotechnology, Chonbuk National University Jeonju 561-756 Republic of Korea; Vivacious – Centre for Nanoscience and Education Research Lucknow-226020 India; All India Institute of Medical Sciences Rishikesh-249201 India; Department of Applied Chemistry, Babasaheb Bhimrao Ambedkar University Lucknow-226025 India

## Abstract

Nanoparticles having strong optical and electronic properties are the most widely used materials in sensor development. Since the target analyte interacts directly with the surface of the material, the choice of ligand for functionalizing the surface of the material is the key for its further applications. The functionalized surface of the material makes it suitable for required applications as it controls the size of the particle during its growth from the solution phase. Biomolecule capped nanomaterials are favourable for various applications in bio-sensing. In the present work, an attempt has been made to explore the biologically active molecule imidazole as capping agent for ZnS semiconductor nanoparticles or quantum dots (QDs). This work explores the possibility of replacing conventional thiol-zinc bonding and hence paves new pathways for biomolecules having the possibility of being efficient capping agents. Computational chemistry has been used to study the mechanism of bonding between one of the nitrogen atoms of imidazole and the zinc ion of the ZnS QDs. The quantum chemical insight not only explores the most spontaneous interaction of zinc ion and imidazole molecule so as to act as an efficient capping agent but also explains the probable bonding site for nitrogen–zinc chemistry. The tailormade Mn doped ZnS QDs are one of the most promising materials for probe and sensor development. The ZnS core having non-toxicity and the emission in longer wavelength due to manganese makes this material highly useful biologically. The aqueous route of synthesis has been employed to obtain a highly homogeneous and pure material which was further characterized by UV (Ultra Violet spectroscopy), Spectrofluorometer, Transmission Electron Microscope and X-ray Diffraction. The toxicity at the cellular and genetic levels was also investigated to prove the potential of the imidazole capped Mn doped ZnS QD as a biocompatible material.

## Abstract 2

Nanoparticles having strong optical and electronic properties are the most widely used materials in sensor development.

## Graphical abstract

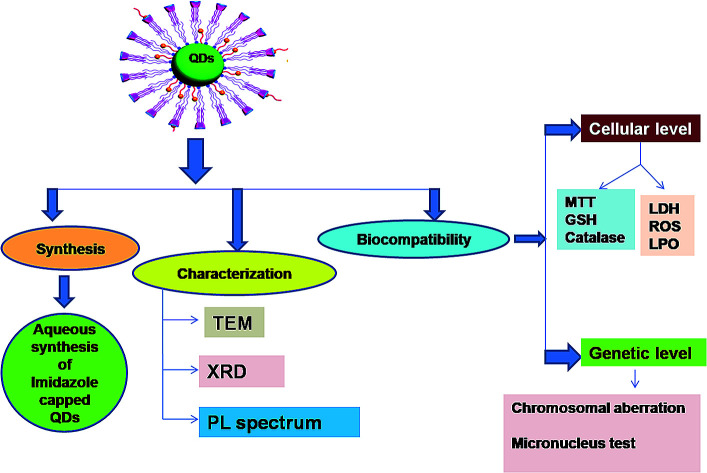

## Introduction

The evolution of functionalized materials in smaller dimensions has revolutionized the nanotechnology world with their unique properties. Among various nanomaterials, quantum dots (QDs), due to their small size, ease of functionalization, stability and tunable optical properties, are key materials that have been extensively studied for imaging and sensing applications.^1–3^ The core material of QDs very commonly comprises of oxides or sulphides of zinc, cadmium and mercury belonging to group II of the transition metals.^4^ For the application of nanoparticles in biological fields they have to pass through the toxicity tests.^5^ Hence toxicity assessment is a prerequisite for the application of QDs in living cells. The toxicity of the QDs depends on various parameters like core material, capping agent and synthetic route. Cadmium based QDs have shown toxicity at all levels due to leakage of the metal ion in the matrix.^6^ Although the emergence of zinc sulphide/cadmium sulphide core–shell QDs solved many of the problems of core containing material but there is still a need for some more advancement in the development of biocompatible nanoparticles.^7^ Thus the use of biomolecules as surfactants will help in extending the research of obtaining non-toxic and highly applicable QDs from the zinc sulphide core.^8^ Further, in order to enhance the optical properties of the ZnS QDs, doping of the ZnS lattice with manganese has been routinely done which gives longer wavelength emission spectra in the orange region of the electromagnetic spectrum and the material is also non-toxic in nature.^9^ Thus ZnS core doped with manganese and capped with a suitable molecule QDs have paved the pathway to using biologically active materials in nanotechnology research.^10^

Various routes have been investigated to synthesize small sized, homogeneous QDs but among all existing methods, aqueous synthesis of the particles involving low temperature and greener methodologies has various advantages over the conventional ones.^10^ The recent advances in the synthetic chemistry of ZnS QDs involving surfactants or capping agents for controlling the growth is expected to lead to the existence of newly generated advanced nanomaterials with highly improved quality.^11^

Developing new capping agents that can reduce the toxic effects, improve the optical properties and also control the growth of quantum dots is a highly challenging task.^12^ Capping agents are very significant as they are the only part of the nanomaterial that interacts with the surface to the target molecule and helps in sensing the analyte with high selectivity and sensitivity.^13^ The metal ion of the nanomaterial acts like a Lewis acid and hence the target ion acting like Lewis base interacts with the metal ion forming a strong covalent bond. The functional group of the capping agent gives the whole species an ability to get functionalized for water solubility and biocompatibility.^14^ Conventionally, thiol containing capping agents are used to attach with the metal ion, but this leaves us with limited molecules to be used for controlling the Ostwald growth.^15^ Also sensing of metal ions becomes difficult if thiol group is blocked upon attaching the capping agent to the core metal of the host crystal lattice. There are various capping agents that have been explored, like mercaptophenylboronic,^16^ thioglycerol, l-cysteine,^10^ MPA (mercaptopropionic acid), MPTS (mercapto propyl trimethoxysilane),^13^*N*-acetyl l-cysteine, glutathione,^17^ thioglycolic acid, cysteamine, *etc.* QDs based bio-conjugates are also developed for detection at high sensitivity.^18^ The physical properties of QDs make it a very strong material in electroluminescence sensing and other sensing mechanisms that involve optoelectronic properties.^19–23^ Recent years have also explored core–shell QDs capped with polymers or shells for sensing macrobiomolecules.^24^ Mostly the conventional capping agents have mercapto as donor site for attachment to the metal. Hence it is necessary to exploit other ligands, like nitrogen, to develop capping agents having nitrogen as donor atom.^25^ Imidazole molecule having two nitrogen atoms in the ring can serve as a ligand to donate its electron pair or charge to the zinc atom.^26^

Herein we have reported for the first time the imidazole capped QDs along with its toxicity profiling. The imidazole containing molecules are a major source of interest for chemists as well as for biologists since many compounds with imidazole or imidazole derivatives are used for pharmaceutical applications.^27^ The imidazole molecule is a planar 5-membered ring with 3C and 2N atom in conjugation. It is classified as a diazole heterocyclic aromatic molecule that is soluble in water and other polar solvents. Many natural products, especially alkaloids and other biological molecules (histidine) are having the imidazole ring. Imidazole group of drugs are commonly used as antifungal drugs. Nitrogen atom of the imidazole ring binds to the fungal cytochrome P450 enzyme and inhibits the conversion of lanosterol to ergosterol by inhibiting lanosterol 14-α-demethylase, thereby disrupting fungal cell membrane. Imidazoles are broad spectrum antifungals especially effective against Candida species. Topical antifungals like clotrimazole, miconazole *etc.* are effective against oral and vulvovaginal candidiasis. Ketoconazole is particularly used against systemic fungal infections. A recent study has demonstrated the utility of controlled release nanoparticle linked clotrimazole for vaginal candidiasis.^28^ Synthetic imidazole have many potential pharmacological uses.^29^ Imidazoles due to diverse synthetic properties with varying physical and chemical properties are very exciting surfactant that can be used with living cells.^30^

Zinc has a tendency to form bond with nitrogen atom of the imidazole ligand due to the basic character of imidazole and acid character of zinc ion enabling complex formation.^31^ The interaction of nitrogen of histidine and zinc was well established and have been used in sensing dopamine by Ravi Arunan *et al.*^38^ This feature of zinc was employed to synthesize and explore Mn:ZnS QDs capped with imidazole.

In the present work, for the first time an attempt was made to obtain highly efficient optical probe with low toxicity. The preference of the capping agent is based on the adaptability of imidazole, its water solubility and strong connectivity with biological world. As of our acquaintance, it is the foremost report of the synthesis and toxicological scrutiny of imidazole Mn:ZnS capped quantum dot. We have also performed computational studies to understand the mechanism of bonding between nitrogen atom of imidazole and zinc ion of the ZnS QDs.^32–37^ The synthesized QDs capped with imidazole ring containing nitrogen as heteroatom will have potential to be used in bio-mimicking where the enzymes like carbonic anhydrase can be mimicked by using zinc as the metal ion and imidazole as ligand.

## Materials and methods

### Computational details

The quantum chemical calculations to study the interaction of imidazole with ZnS have been done *via* the density functional theory (DFT). Structure of imidazole and Zn^2+^ (generate from ZnS in aqueous medium) and products obtained after reactions have been fully optimized to minimum energy at B3LYP/6-311++G (d, p) theoretical model. The functional B3LYP consist of Becke 88 exchange functional and correlation functional of Lee, Yang and Parr.^32,33^ This is the most widely used functional and is able to reproduce experimental results very accurately. The basis set employed here is adequate for the elements which are present in the current reaction system. To ensure that all the structures are minima, analytical regularity calculation has been done at the same level of theory. All the energy minima have been found to have only positive frequencies. Same set of analytical frequencies has been used to calculate zero point vibrational energy corrections. Further, electronic energies of all the structures has been corrected by performing calculations at single point with CCSD (T)/6-311++G (d, p) level of theory.^34,35^ No transition state (TS) has been observed in this work. The barrier less nature of the reactions has been confirmed by performing constraint optimizations along proposed reaction coordinate. Gaussian 09 was used to perform all calculations of electronic structure.^36^

### Analytical grade reagents

Analytical grade reagents and chemicals were used in the experiments unless otherwise stated. Milli Q water (water deionized and double distilled) was used in every part of the experiments. Zinc sulphate heptahydrate (ZnSO_4_·7H_2_O), manganese(ii) chloride tetrahydrate (MnCl_2_·4H_2_O), l-cysteine (97% purity), imidazole and ethanol be procured from Sigma Aldrich. Sodium sulphide (Na_2_S·*X*H_2_O) was procured from SD Fine Chemicals (Mumbai). Milli-Q water was used to prepare the stock solutions and stored at 4 °C until their usage. All the particular biological assay chemicals and reagents be purchased from Sigma (Sigma St. Louis, MO, USA) unless otherwise stated. Dulbecco's Modified Eagle Medium/Nutrient Mixture F-12 (DMEM/F-12) was used as culture medium, antibiotics, fetal bovine serum (FBS) and trypsin–EDTA were procured from Gibco BRL, USA. The plastic wares, Culture wares and other material used in the study were purchased commercially from Nunc, Denmark.

#### Cell culture

In the present study we have used cultured Human lung A549 cell line which was procured from American Type Culture Collection (ATCC) Manassas, USA as per the standard protocols. The cells were maintained at Department of Animal Science and Biotechnology, Chonbuk National University, Jeonju, Korea Republic. The methodology of culture was based on earlier reports of Tripathi V. K. *et al.* 2014.

#### Synthesis of imidazole capped Mn:ZnS QDs

In a three necked flask of 100 ml, 10 mM of ZnSO_4_·7H_2_O, 1.5 mM MnCl_2_·4H_2_O and water 100 ml were added with stirring for 20 min in presence of nitrogen gas. To control the growth 10 mM imidazole dissolved in water was added. The pH of the resultant mixture was maintained at 10. After being stirred rigorously for 20 minutes, 10 mM of Na_2_S was injected in to the mixture. The temperature for 1 hour of the final reaction was maintained at 70 °C. For ageing, the mixture was continued stirring for 18 hours at room temperature. Finally a precipitate of QD capped with imidazole was obtained which was washed further with ethanol & water through centrifugation and then dried under vacuum. The finally obtained QDs were stored in water as a suspension. Thus good quality imidazole Mn:ZnS capped QDs, a water dispersible, highly purified are obtained.

#### Characterization of the synthesized QDs

Using transmission electron microscopy (TEM, Tecnai-G2-SPIRIT FEI, Netherland) the size of the nanocrystal was observed. A single drop of aqueous solution was placed on the copper grid to obtain images of QDs after sonication and coated with formvar. Using a spectrophotometer, spectrophotometer (SPECORD 210 PLUS, Analytik Jena, Germany) absorbance and band gap energy was obtained. LS 55 photoluminescence spectrometer, PerkinElmer, UK, was used to obtain fluorescence spectra of the QDs. The wavelength of 340 nm was used for excitation to obtain an emission wavelength that corresponds to 590 nm. The powder XRD pattern was performed by the Cu target source (SEIFERT, Germany) at ACMS Lab, IIT Kanpur, India. The FTIR spectra of pure imidazole and imidazole capped QDs were carried out on PerkinElmer spectrum RXI instrument.

#### Toxicity evaluation assay

##### MTT assay

Following the protocol of Tripathi V. K. *et al.* 2014, Cytotoxicity assessment was done using standard endpoint *i.e.*, tetrazolium bromide MTT assay. In concise, A549 cells (1 × 10^4^ cells per well) were incubated in the CO_2_ incubator for 24 h at 37 °C and seeded in 96-well tissue culture plates. Cells were then exposed to medium under high humid conditions containing uncapped QDs (0.025, 0.05, 0.1, 0.2 and 0.4 g l^−1^) and capped QDs (0.2, 0.4, 0.8, 1.0 and 2.0 g l^−1^) for 24–96 h at 37 °C in 5% CO_2_-95% atmosphere. Prior to the completion of respective incubation periods, tetrazolium salt (10 μl per well; 5 mg ml^−1^ of stock in phosphate-buffered saline [PBS]) was added 4 h. At the completion of the incubation period, 200 μl of culture grade dimethyl sulfoxide was added to each well. The content was mixed well until dissolved completely. Using multi-well microplate reader (Synergy HT; Bio-Tek, USA) plates were then incubated for 10 min at room temperature and color was read at 550 nm. To maintain basal control the unexposed sets were also run parallel under identical conditions.

#### Neutral red uptake assay

In the present study the protocol described earlier by Kumar V. (2015) was followed to carry out the assay. In an identical experimental setup as to MTT assay the cells were exposed to uncapped and capped QDs. After the completion of the incubation period, NRU salt (50 μM ml^−1^ in the medium) was added in composition of 100 μl per well plate and further incubated for 3 h. After this the reaction mixture was carefully taken out and plates were washed with washing solution (100 μl per well) containing 1% CaCl_2_ (w/v) and 0.5% HCHO (v/v) to get rid of unincorporated dye. After removing the washing solution a mixture was added in composition of 200 μl 1% acetic acid and 50% ethanol. Using multi-well micro plate reader (Synergy HT, Bio-Tek, USA) the plates were kept on rocker shaker for 10 min at room temperature and then analyzed at 540 nm. To serve as control the unexposed sets were also run under identical condition.

### Lactate dehydrogenase release assay

LDH release assays a reliable, nonradiative technique for analysis of cytotoxic lytic activity. LDH release assay is a method to measure the membrane integrity as a function of the amount of cytoplasmic LDH released into the medium. Using ready-made commercially available LDH assay kit for *in vitro* cytotoxicity evaluation (TOX-7, Sigma St. Louis, MO, USA) the assay was carried out. By the action of LDH, reduction of NAD take place in the method. In the stoichiometric conversion of a tetrazolium dye the reduced NAD (NADH^+^) was utilized. Using the multi-well plate reader the colored compound was measured at wavelengths 490 and 690 nm. The cells were processed for LDH release assay similar to MTT assay as the cells were exposed to 0.025, 0.05, 0.1, 0.2 and 0.4 g l^−1^ of uncapped QDs and 0.2, 0.4, 0.8, 1.0 and 2.0 g l^−1^ capped QDs for different time periods after the completion of the respective time periods. As per the experimental schedule culture plates were removed from CO_2_ incubator and centrifuged at 250×*g* for 4 min. Then supernatant of each well was transferred to a flat bottom 96 well culture plate and processed further for enzymatic analysis as per the instructions.

#### Oxidative stress studies

##### Reactive oxygen species (ROS) generation

ROS are highly reactive molecules to oxidize cell. In this analysis, according to method of Kashyap M. P. *et al.* 2011, uncapped and capped QDs induced ROS generation was assessed in A549 cells using 2′,7′-dichlorodihydrofluorescein diacetate (DCFH-DA, Sigma Aldrich) dye as fluorescence agent. Through fluorometric analysis ROS generation was studied.

##### Glutathione (GSH) levels

Glutathione (GSH) levels deficiency put the cell at the risk for oxidative damage so this method were assessed using commercially available kit (ApoGSH Glutathione Colorimetric Assay Kit, Catalog No. #K261-100, Biovision, USA). In brief, using multi-well microplate Reader (Synergy HT, Bio-Tek, USA) the deproteinated samples were transferred to 96 well plates (50 μl per well), mixed with 150 μl freshly prepared assay cocktail and read at 405 nm at 5 min intervals up to 30 min. To calculate the experimental values standard curve was plotted using the glutathione standard supplied in the kit.

##### Lipid peroxidation (LPO)

LPO is the oxidative degradation of lipids resulting in cell damage. For analysis, commercially available kit (Lipid Peroxidation Assay Kit, catalog no. 705002; Cayman Chemicals, USA) as per the manufacturer's protocol used for the exposure of uncapped QDs (0.025, 0.05, 0.1, 0.2 and 0.4 g l^−1^) and (0.2, 0.4, 0.8, 1.0 and 2.0 g l^−1^) capped QDs for 6, 12 and 24 h. The cells were harvested by centrifugation at 1000 rpm for 10 min and processed for the estimation of lipid peroxidation.

##### Catalase (CAT) levels

Catalase is an enzyme, protein that catalyzes chemical reactions and protect cell from oxidative damage by ROS. Activity was similar to the LPO for catalase analysis. Activity was measured using commercially available kit for catalase activity (Catalog no. 707002; Cayman Chemicals, USA).

#### Genotoxicity studies

##### Micronucleus (MN) assay

*In vitro* micronucleus assay was carried out using standard protocols Kashyap M. P. *et al.* 2010 for toxicological screening for genotoxic compounds. The micronucleus (MNs) analysis, was performed by 1 × 10^5^ A549-cells which were seeded in the each well of a six well tissue culture plates (Nunc.) and was further allowed to adhere for 24 h in CO_2_ incubator at 37 °C. The exposure given to the cells were of varied concentration for 24 h. The uncapped QDs were exposed to concentration 0.025, 0.05, 0.1, 0.2 and 0.4 (g l^−1^) and capped were exposed to 0.2, 0.4, 0.8, 1.0 and 2.0 (g l^−1^). Washing and supplementing of the cells was done with cytochalasin B (3 μg ml^−1^, Sigma) containing medium. Incubation time was kept as 24 h. A hypotonic buffer (0.075 M KCl) for 5–10 min at 37 °C was used for harvesting and Carnoy's fixative (methanol/acetic acid, 3 : 1) was used for fixation of the cells. To get good imaging cells were dropped in a proper orientation onto the slides and stained with 5% Giemsa in phosphate buffer for which pH: 6.8 was stabilized for 15–20 min. The mounting with DPX was finally done for microscopic examination. Using a Nikon Eclipse 80i upright microscope attached to a Nikon digital CCD cool camera (Model DS-Ri1 of 12.7 megapixel) a minimum of 1000 binucleated cells with well-defined cytoplasm in each slide was scored for the presence of micronucleus. Mean of threeslides was evaluated for presenting the data.

##### Chromosomal aberration (CA) assay

The CA assay was carried out using standard protocols Kapoor E *et al.* 2014 to identify the toxicological screening of genotoxicity. The cells that were cultured for the assay was A549 cells. The cells were exposed to different concentration of uncapped and capped QDs for 24 h. To harvest the cells, colcemid (0.15 μg ml^−1^ final concentration) was added and then kept for 4 h. The cells were washed with Hank's balanced salt solution. Later the cells were given a hypotonic shock in potassium citrate (0.8%) for 30 min. Cold fixative (methanol : acetic acid, 5 : 2 ratios) was used for fixing the cells. Cells were stained in 5% Gurr's Giemsa on a clear slide. Using a Nikon Eclipse 80i upright microscope attached to a Nikon digital CCD cool camera (Model DS-Ri1 of 12.7 Megapixel) the cells were scored for the presence of CA. Mean of three slides was taken for presenting the final data.

##### Statistical analysis

For statistical analysis at least three experiments were conducted and the data was expressed as mean and standard error of means (mean ± SE). Thus,*P < 0.05 was taken to indicate significant differences. Softwares like ANOVA which was also followed by *post hoc* Dunnett's test was employed to detect differences between the groups of treated and control.

## Results and discussions

### Experimental work

The aqueous route of synthesis was involved to obtain the luminescent manganese doped zinc sulphide QDs. The precursors used in the study was zinc sulphate salt, manganese salt while sodium sulphide was used to provide sulphide ions for the Mn doped ZnS nanomaterial. The imidazole molecule has two nitrogen atom one having N–H bond and other having lone pair. The synthesis was performed in water and further change to pH was done with NaOH. The base attacks on the H atom attached to the nitrogen atom and this result in the formation of nitrogen metal bond by the influence of negative charge on the first nitrogen atom. The deprotonation of imidazole was done to obtain highly basic anion. The capping agent was added in appropriate amount to reduce the formation of only metal ligand complex. The imidazole bonded zinc was further reacted with sulphide ion. There is always a competition between capping agent and anion hence controlling this aspect is crucial. To overcome this, fast addition of sulphide ion was done to form zinc sulphide precipitate immediately. After addition of sulphide ion the temperature of the reaction mixture was increased for 1 hour. As the temperature increases the rate of ZnS formation increases as well. Synthesis of ZnS nanoparticle as a result involves another step where the formed precipitate undergoes ageing. The ageing of the nanomaterial has significant role in its crystallinity. Here room temperature was chosen for growing the particles. Subsequent to 18 h of ageing the material was washed with ethanol and water several times and final mixture was kept on oven for drying. The synthesis resulted in imidazole capped Mn doped zinc sulphide quantum dots following aqueous route of synthesis.

**Fig. 1:**
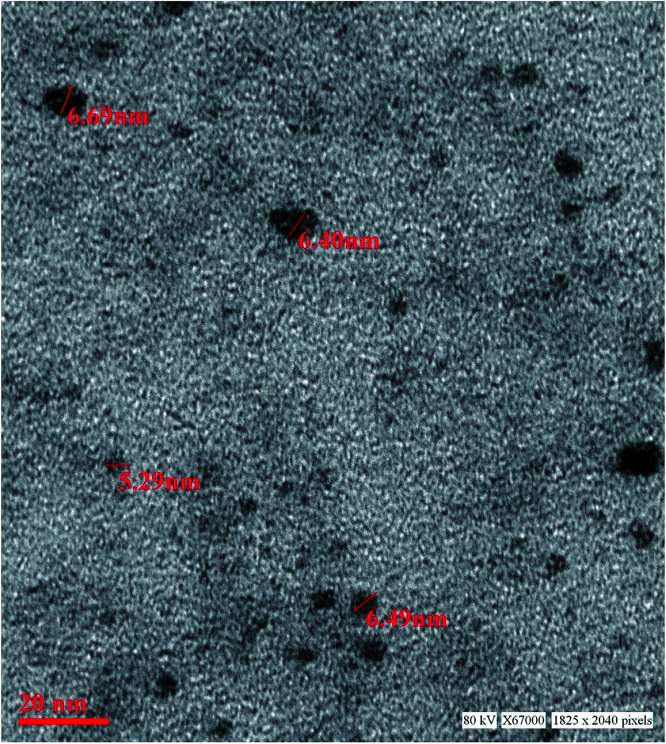
TEM image of the synthesized imidazole capped QDs.

The as synthesized material obtained was characterized further by TEM for particle size determination as shown in the Fig. 1. The particles in the size range 5.5–6.5 nm were obtained. The TEM results show that homogeneous particles have been synthesized. The typical particle size of the material is below 10 nm hence it is inferred that QD have been obtained. The dark image of the material is due to the zinc metal which is classified as heavy metal. The strong transmission of the material confirms the constituent of the material under investigation. There is no clump or cluster in the image which proves that imidazole acts as good capping agent and controls the particle size effectively.

**Fig. 2:**
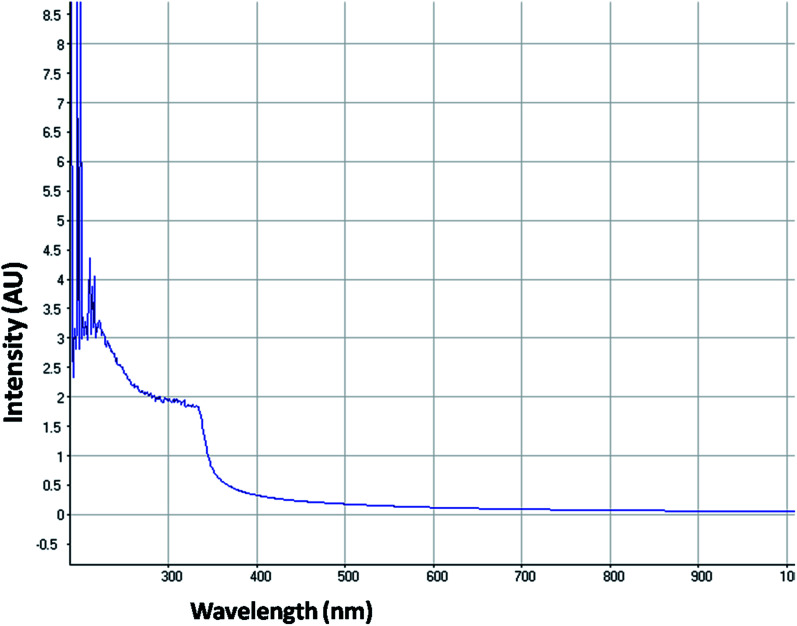
UV-visible absorption spectrum of imidazole capped Mn:ZnS QDs.

**Fig. 3:**
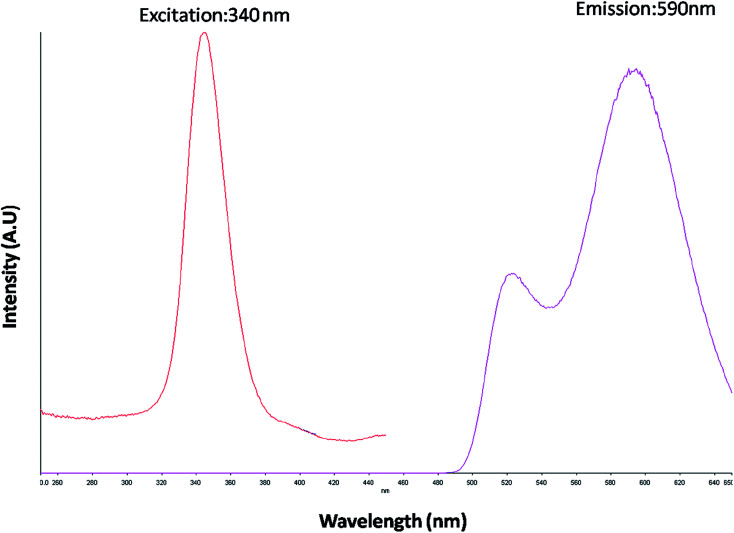
PL emission and excitation spectrum of imidazole capped Mn:ZnS QDs.

**Fig. 4:**
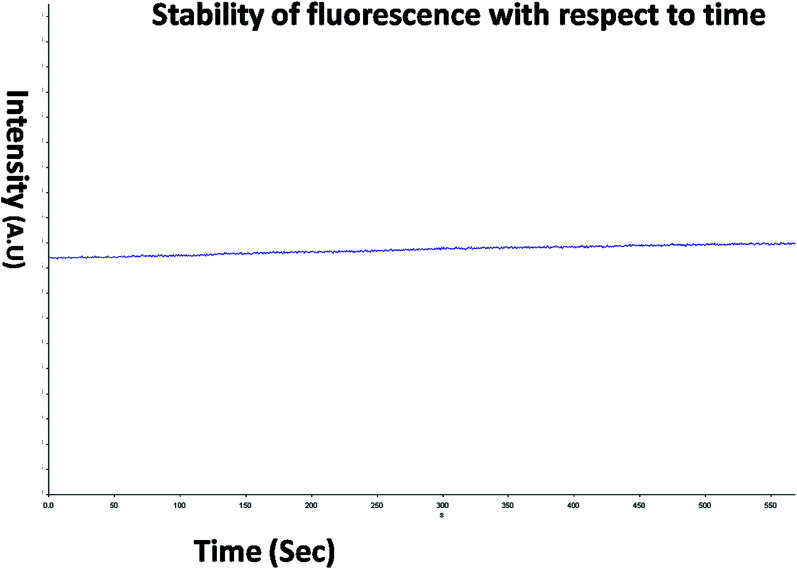
PL stability of the QDs from 0 to 550 seconds.

The optical property of the as synthesized QDs was performed by UV and spectrofluorometer. The absorbance at 310 nm and cut off at 340 nm as shown in Fig. 2, proves the blue shift. The cutoff at 340 nm also supports the photoluminescence spectra (Fig. 3). The photoluminescence spectra show that the excitation of the QD is 340 nm and emission is at 590 nm. The strong quantum confinement is there in the band gap due to small sized QD. The emission at 590 nm confirms that manganese ion has been doped in the crystal lattice. The trap states created due to the manganese ion results in higher emission wavelength. Thus the material with highly enhanced optical property has been synthesized. Both UV and photoluminescence spectra support the band gap (3.6 eV), excitation (340 nm) and emission (590 nm) parameter. Hence imidazole molecule had not altered the optical property of the synthesized quantum dots. The optical stability of the synthesized QD was evaluated as well, which shows a stable photoluminescence for a significant 550 seconds (Fig. 4). Thus the luminescence intensity of the imidazole capped QD do not decrease with respect to time and hence highly efficient probe was synthesized which can be used for a longer time in imaging.

**Fig. 5:**
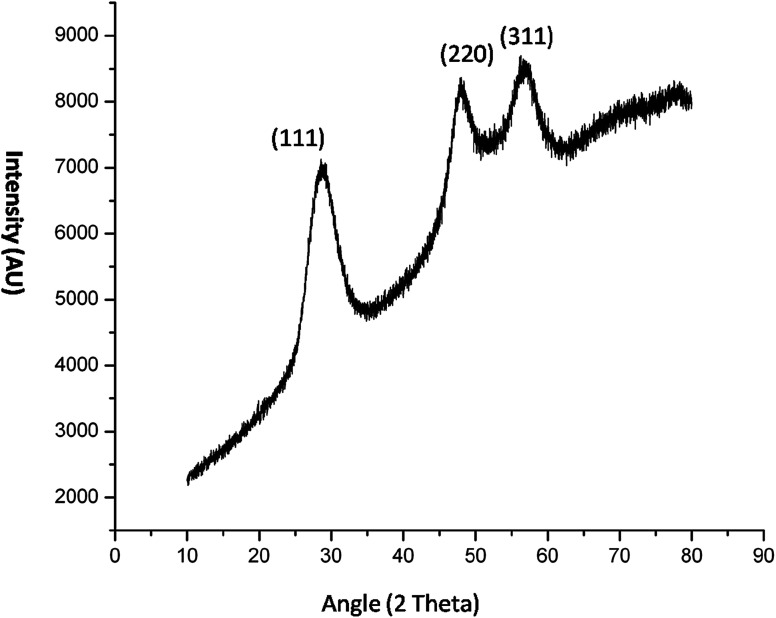
XRD pattern of imidazole capped Mn:ZnS QDs.

The crystal property of the material was evaluated by XRD measurement. It was observed in the diffraction pattern that high intensity peaks were observed at 28.47 and 56. The XRD pattern (Fig. 5) shows miler planes at (111), (220) and (311) which is in accordance with JCPDS no. 05-0566. The lattice parameters of the QD synthesized and library is similar which confirms the zinc sulphide lattice of the type cubic zinc blende. The purity of the material can be acknowledged by having no additional peaks in the diffraction pattern. The peaks in the diffraction pattern shows that they are of high intensity which attribute to the high crystalline property of the QDs. The dimension of the imidazole capped QD was 9 nm. The smaller size has been also confirmed by the XRD data since the peaks are broaden. The size of the material was calculated by Scherrer equation *β*_1/2_ = 0.94*λ*/*d* cos *θ* (Liu *et al.* 2010).^12^ The peak width shows the characteristics of ZnS QDs. Further the capping of imidazole with Mn doped ZnS QDs were confirmed by analyzing FTIR bands. The decrease in intensity of N–H band (Fig. 16b) (3416 cm^−1^) shows strong interaction of N–H bond with the zinc ion. Although other bands like C–H stretching (2931–3132 cm^−1^) shows lower intensity in the imidazole capped QDs (Fig. 16b).

Hence high quality manganese doped zinc sulphide QD have been synthesized. It is inferred that the imidazole controls the size of the material without affecting the chemical property and material property. Here we have also evaluated the biological applicability of the capped QD with respect to uncapped one.

### Computational insight to explain imidazole–zinc bonding

**Fig. 6:**
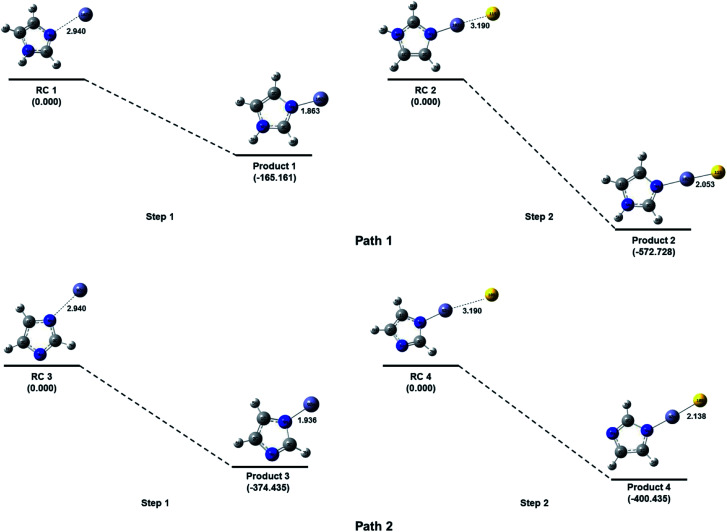
Energy level diagram (at CCSD(T)/6-311++G(d, p)//B3LYP/6-311++G(d, p) level) for the formation of imidazole capped Mn:ZnS QDs without basic medium (path 1) and with basic medium (path 2). Relative energies are calculated with respect to the separated reactants in each step. All the bond lengths are shown in angstrom.

Imidazole is a five membered ring with both basic and acidic nature due to the presence of lone pair on the nitrogen atom and NH group. Here in the quantum chemical study with the help of computational simulation following density functional theory we have studied the formation of zinc imidazole complex following two approaches. In the first approach we have modeled the reaction pathway of zinc ion with imidazole molecule. To model the reaction, Zn ion is placed in the plane of imidazole ring at 2.940 Å (sum of van der Walls radius of Zn and N) from the nitrogen atom (other than NH). This initial structure is optimized to minimum energy and we obtain the Zn–imidazole complex (hereafter product 1). A lone pair of electrons is present on this nitrogen atom of the imidazole molecule which is donated to zinc ion resulting in the formation of this complex (path 1, Fig. 6). This step is barrier less and exothermic with the enthalpy of formation (Δ*H*) is −165.1605 kcal mol^−1^ with respect to the separated reactants. Product 1 now carries a positive charge and is susceptible to the nucleophilic attack. The S^2−^ which is added after the formation of product 1 in the reaction system, now quickly react and form a bond with Zn–imidazole complex producing imidazole capped ZnS (product 2). This reaction is exothermic with enthalpy of formation being −572.7279 kcal mol^−1^.

Further, in the similar way the addition of Zn^2+^ to the NH group of imidazole was also attempted by placing Zn^2+^ at 109.5° with imidazole plane and at 2.940 Å from the nitrogen atom of the NH group. However, the zinc ion was not approaching towards the NH bond for the bond formation during optimization of the reaction system. The probable reason for this phenomenon could be the unavailability of electrons on the nitrogen of NH functional group as compared to the other nitrogen of the imidazole ring. The addition of Zn^2+^ on this nitrogen (*i.e.* of NH) can be feasible if a high electron density could be possible on the group which can interact with the zinc ion. Here in we have removed the H of N–H bond by reacting imidazole molecule with base as described in the experimental section which results in the formation of the imidazole anion. The imidazole anion is a symmetric heterocyclic ring with a uni-negative charge which is distributed among all the atoms in the ring. To study the interaction of imidazole anion with Zn^2+^, it is placed at 2.940 Å from nitrogen of the ring in the same plane as imidazole (Fig. 6). Optimization of this initial structure results in the product 3 which forms barrierlessly with enthalpy of formation −374.4349 kcal mol^−1^. It will further react barrierlessly with sulfide ion (S^2−^) to produce product 4 (with Δ*H* = −400.7276 kcal mol^−1^).

The above results shows that when reaction of imidazole and Zn^2+^ occur within the basic medium, its efficiency increases, (Δ*H* = −165.1610 kcal mol^−1^ without basic medium, and Δ*H* = −374.4351 kcal mol^−1^ in basic medium). The first step in path 1 is the ion-molecule interaction which is less efficient compared to first step in path 2 where the interaction is between imidazole ion and Zn^2+^ ion, which is an ionic interaction with respect to both of the reactants therefore this should be spontaneous. The product 4 has 313.3782 kcal mol^−1^ less energy than product 2. Due to symmetric behavior of imidazolium anion there is equal probability of attack of zinc ion towards both of the nitrogen of the imidazolium anion. The pathway 2 is more efficient thermodynamically than pathway 1 (total Δ*H* = −737.8884 kcal mol^−1^ without basic medium, and Δ*H* = −775.1625 kcal mol^−1^ in basic medium). Hence it can be argued that pathway 2 is suitable to produce imidazole capped—ZnS quantum dots.

*In vitro* toxicity and genetic toxicology was evaluated by various parameters. Cellular toxicity was evaluated by MTT, NRU, LDH, ROS, catalase, GSH and LPO. The toxicity at genetic level was performed by chromosomal aberration and micronucleus test. *In vitro* toxicity and genetic toxicology was evaluated by various parameters. Cellular toxicity was evaluated by MTT, NRU, LDH, ROS, catalase, GSH and LPO. The toxicity at genetic level was performed by chromosomal aberration and micronucleus test.

### Cytotoxicity assessment

#### MTT assay

**Fig. 7:**
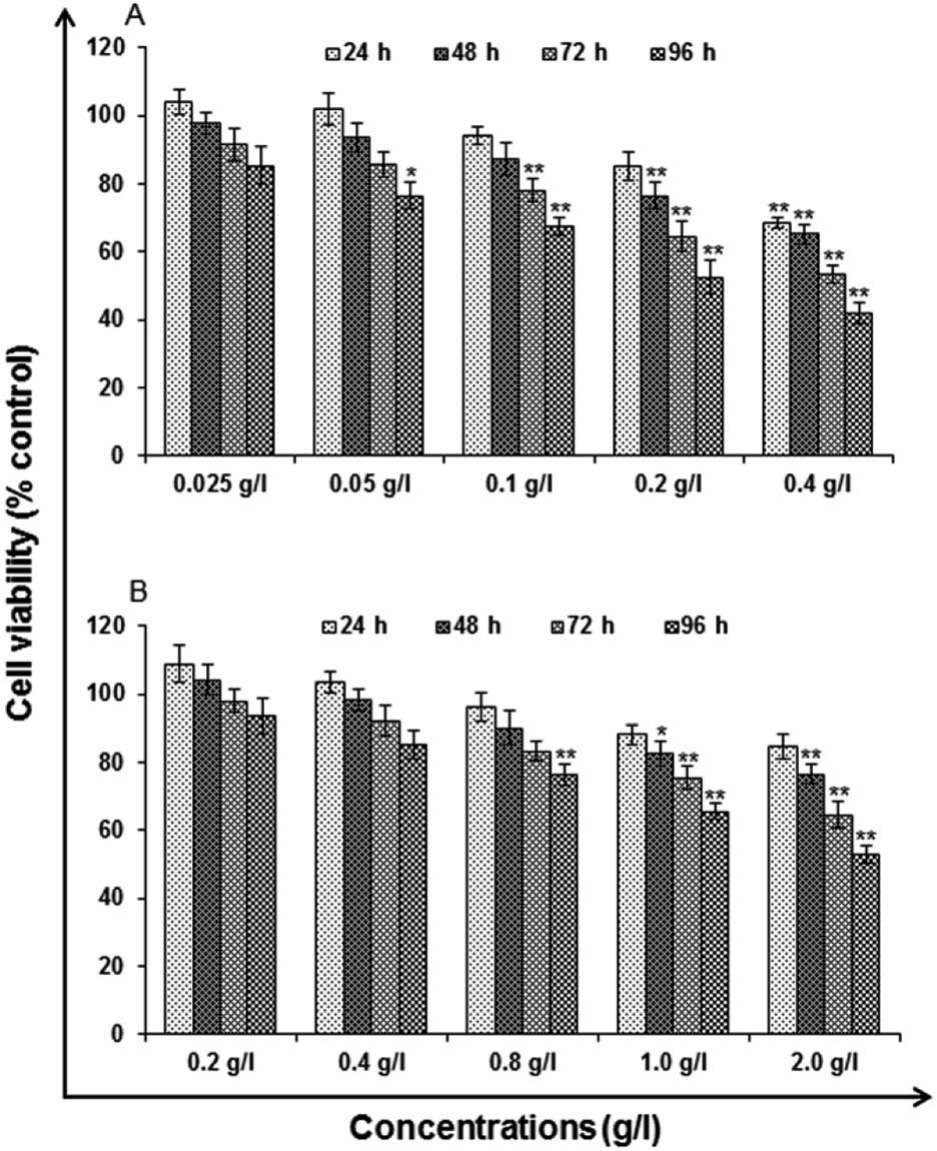
Cytotoxicity/biosafety assessment of (A) uncapped QDs and (B) capped QDs in human lung epithelium cells-A549 (3-[4,5-dimethylthiazol-2-yl]-2,5-diphenyl tetrazolium bromide) assay. Cells were exposed to various concentrations for 24–96 h. The data presented are percent cell viability compared with unexposed control cells. Values are given as mean ± standard error of the data obtained from three independent experiments and each experiment contained at least three replicates. *P < 0.05 = significant.

Results of MTT assay are summarized in Fig. 7. Human lung epithelium cells-A549 responded to uncapped and capped QDs in a dose and time dependent manner. There was no significant reduction in percent cell viability reported all through the exposure period, *i.e.*, till 48 h in the used concentration *i.e.*, 0.025–0.05 g l^−1^ of the uncapped QDs and 0.2–0.4 g l^−1^ of the capped QDs. Whereas, at and above the exposure period of 24 h the concentrations of uncapped used, *i.e.*, 0.1, 0.2 and 0.4 g l^−1^ were found to cause a gradual reduction in percent cell viability, which reaches to significant level. The cell viability reduces to 85.12 ± 4.20, 76.64 ± 3.73, 64.58 ± 4.42 and 52.55 ± 4.79 in the cells exposed to 0.2 g l^−1^ for a period of 24, 48, 72 and 96 h respectively. The reduction was severe in cells exposed to the highest concentration, *i.e.*, 0.4 g l^−1^, where the viability reduces to 68.46 ± 1.69, 65.30 ± 2.83, 53.27 ± 2.60 and 41.92 ± 3.04 at 24, 48, 72 and 96 h respectively, when compared with unexposed control cells (Fig. 7A). Whereas capped QDs show 88.17 ± 2.73, 82.44 ± 3.63, 75.47 ± 3.59 and 65.71 ± 2.3079 in the cells exposed to 1.0 g l^−1^ for a period of 24, 48, 72 and 96 h respectively. The reduction was severe in cells exposed to the highest concentration, *i.e.*, 2.0 g l^−1^, where the viability reduces to 84.53 ± 3.65, 76.51 ± 2.86, 64.57 ± 3.68 and 52.71 ± 2.79 at 24, 48, 72 and 96 h respectively, when compared with unexposed control cells (Fig. 7B).

#### Neutral red uptake assay

**Fig. 8:**
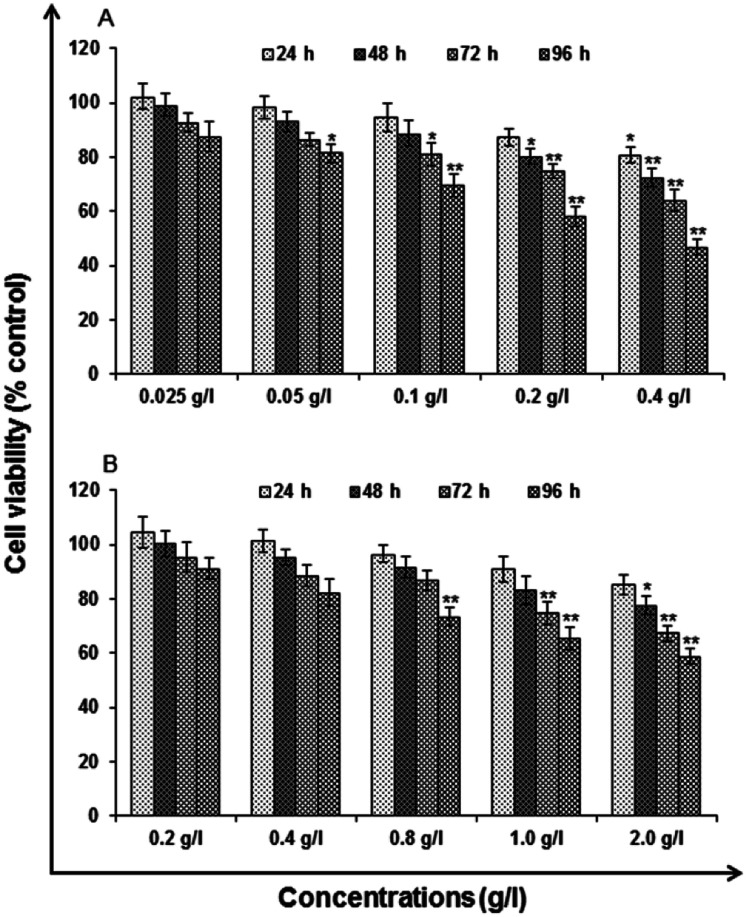
Cytotoxicity/biosafety assessment of (A) uncapped QDs and (B) capped QDs in human lung epithelium cells-A549 (neutral red uptake assay). Cells were exposed to various concentrations for 24–96 h. The data presented are percent cell viability compared with unexposed control cells. Values are given as mean ± standard error of the data obtained from three independent experiments and each experiment contained at least three replicates. *P < 0.05 = significant.

The observations of NRU assay were having similar trends as that to the MTT assay. The lower concentration, *i.e.*, 0.025–0.05 g l^−1^ of uncapped QDs and 0.2–0.4 g l^−1^ of capped QDs were found to induce no significant reduction in percent cell viability, while the higher concentrations, *i.e.*, 0.1, 0.2 and 0.4 g l^−1^ of uncapped and 0.8, 1.0 and 2.0 g l^−1^ of capped QDs were able to induce a dose dependent decrease in the percent cell viability, which reaches to statistically significant levels. The cell viability reduces to 87.31 ± 3.18, 80.17 ± 2.73, 74.86 ± 2.71 and 58.14 ± 3.65 in the cells exposed to 0.2 g l^−1^ for a period of 24, 48, 72 and 96 h respectively. The reduction was severe in cells exposed to the highest concentration, *i.e.*, 0.4 g l^−1^, where the viability reduces to 80.59 ± 2.88, 72.41 ± 3.63, 63.98 ± 3.77 and 46.86 ± 2.68 at 24, 48, 72 and 96 h respectively, when compared with unexposed control cells with respect to uncapped QDs (Fig. 8A). While the capped QDs show 90.77 ± 4.77, 83.12 ± 5.28, 74.83 ± 4.23 and 65.34 ± 4.17 in the cells exposed to 1.0 g l^−1^ for a period of 24, 48, 72 and 96 h respectively. The reduction was severe in cells exposed to the highest concentration, *i.e.*, 2.0 g l^−1^, where the viability reduces to 85.31 ± 3.71, 77.65 ± 3.19, 67.35 ± 2.79 and 58.83 ± 2.72 at 24, 48, 72 and 96 h respectively, when compared with unexposed control cells (Fig. 8B).

#### Lactate dehydrogenase release assay

**Fig. 9:**
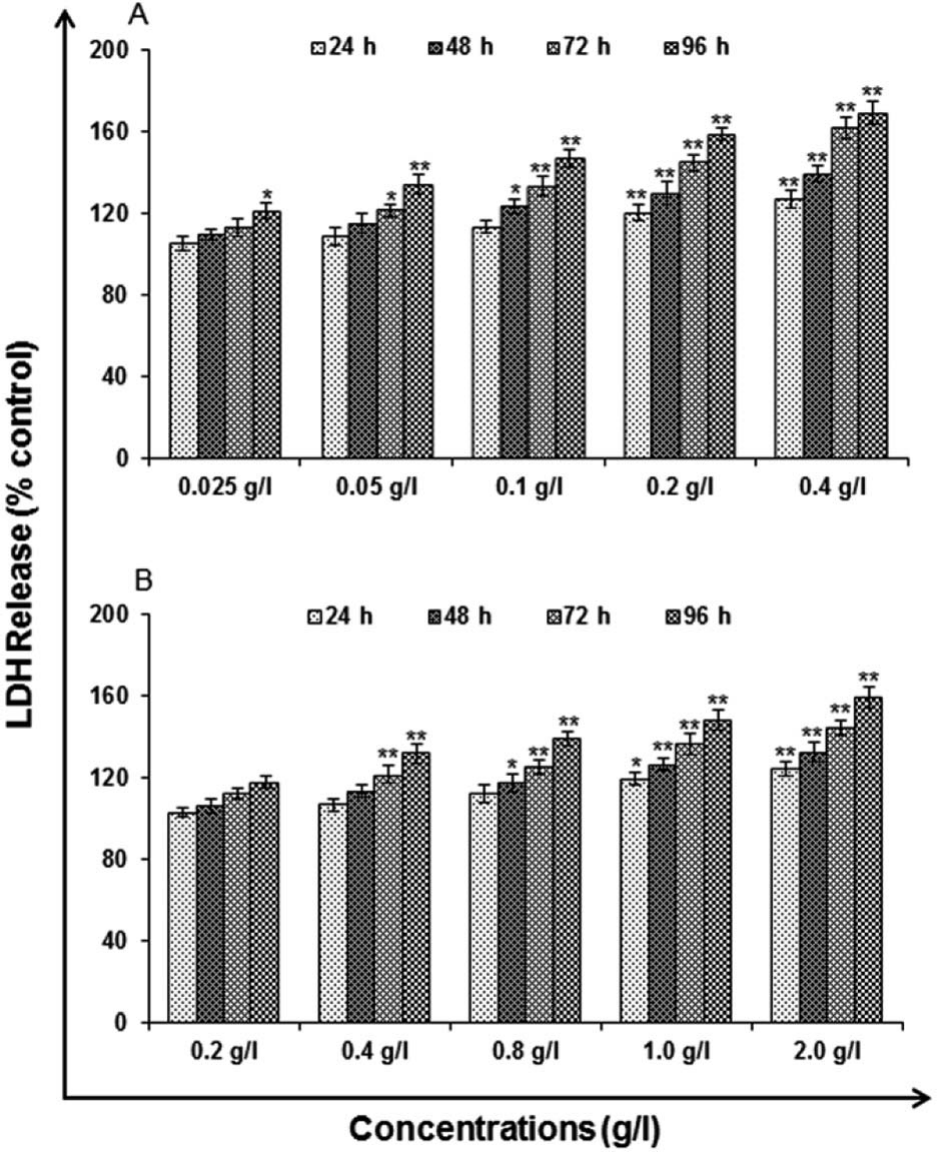
Cytotoxicity/biosafety assessment of (A) uncapped QDs and (B) capped QDs in human lung epithelium cells-A549 (lactate dehydrogenase release assay). Cells were exposed to various concentrations for 24–96 h. The data presented are percent cell viability compared with unexposed control cells. Values are given as mean ± standard error of the data obtained from three independent experiments and each experiment contained at least three replicates. *P < 0.05 = significant.

In Fig. 9A and B, the highlights of the release of LDH subsequent to the exposure of cells for uncapped and capped QDs are presented. There is a significant increase in LDH release for the cells exposed with concentration in the range 0.1–0.4 g l^−1^ of uncapped and 0.8–2.0 g l^−1^ of capped QDs in comparison to unexposed controls. The LDH release was increased significantly to 113.54 ± 2.85, 123.66 ± 3.35, 133.65 ± 5.03 and 147.22 ± 4.34% at 24, 48, 72 and 96 h and it reaches its maximum up to 127.25 ± 4.70, 139.58 ± 3.72, 162.23 ± 5.36 and 169.47 ± 5.85% at 24, 48, 72 and 96 h in cells exposed to 0.1 and 0.4 g l^−1^ concentration of uncapped QDs respectively when compared with unexposed control cells (Fig. 9A). The trends were similar after the exposure of 0.8 g l^−1^ of capped QDs and it shows 112.18 ± 4.16, 117.39 ± 4.46, 125.25 ± 3.33 and 139.49 ± 3.74% at 24, 48, 72 and 96 h which reached up to 124.67 ± 3.71, 132.52 ± 4.69, 144.72 ± 3.66 and 159.16 ± 5.32% at 24, 48, 72 and 96 h after the exposure of 2.0 g l^−1^ when compared with unexposed control cells. No significant changes were found in the cells exposed to 0.025–0.05 g l^−1^ of uncapped and 0.2–0.4 g l^−1^ of capped QDs all through the experiments (Fig. 9B).

#### Reactive oxygen species (ROS) generation

**Fig. 10:**
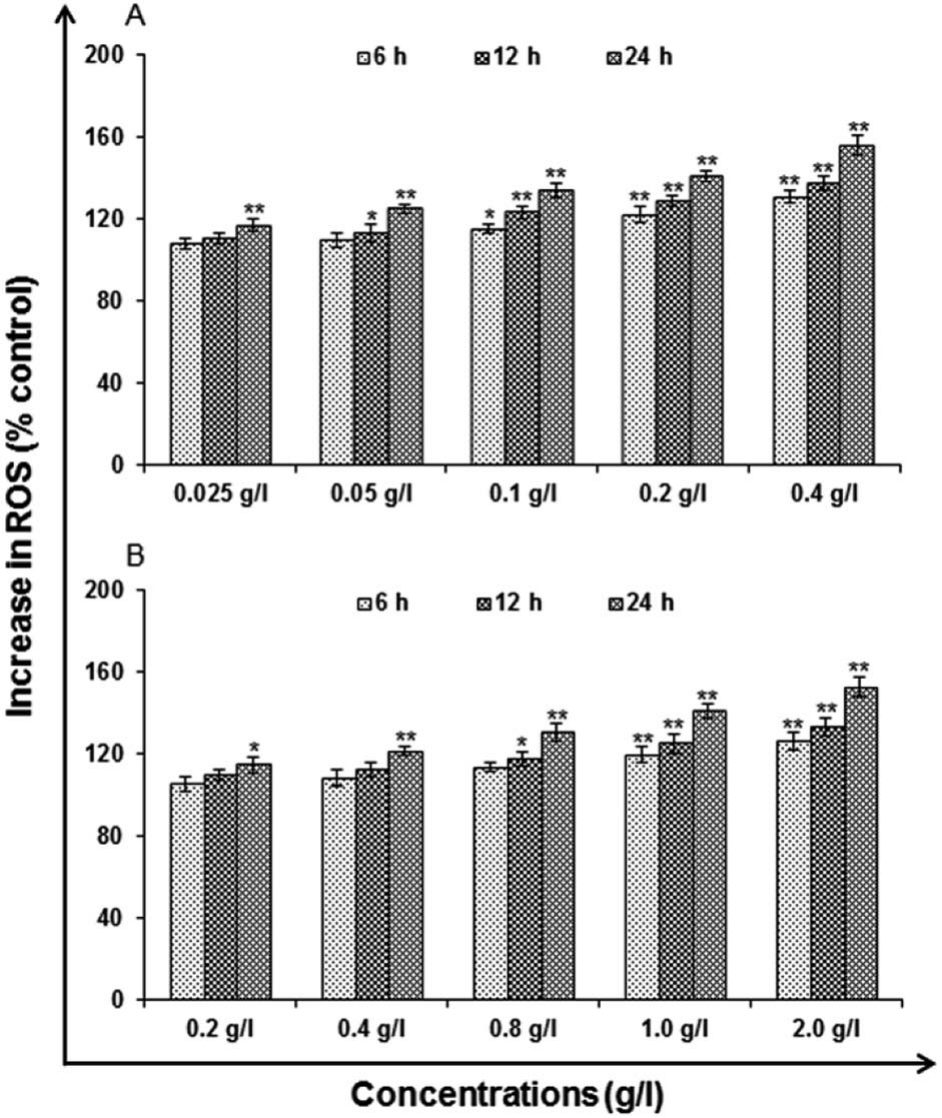
Percent change in ROS generation following 6, 12 and 24 h exposure to various concentrations of (A) uncapped QDs and (B) capped QDs in A549 cells assessed by micro plate reader. Data represented are mean ± SE of three identical experiments made in three replicate. *p < 0.05 = significant.

In Fig. 10A and B the results of uncapped and capped QDs induction and ROS generation are summarized. In the cells it was a significant (*P < 0.05) at 6 h (119.84 ± 3.75, 126.38 ± 4.68), 12 h (125.34 ± 4.80%, 133.56 ± 4.20%) and 24 h (141.23 ± 3.28%, 152.49 ± 4.75%) of exposure. Highest concentration of both uncapped and capped QDs used (0.4 and 2.0 g l^−1^) was affected at every part of the time points.

#### Glutathione (GSH) levels

**Fig. 11:**
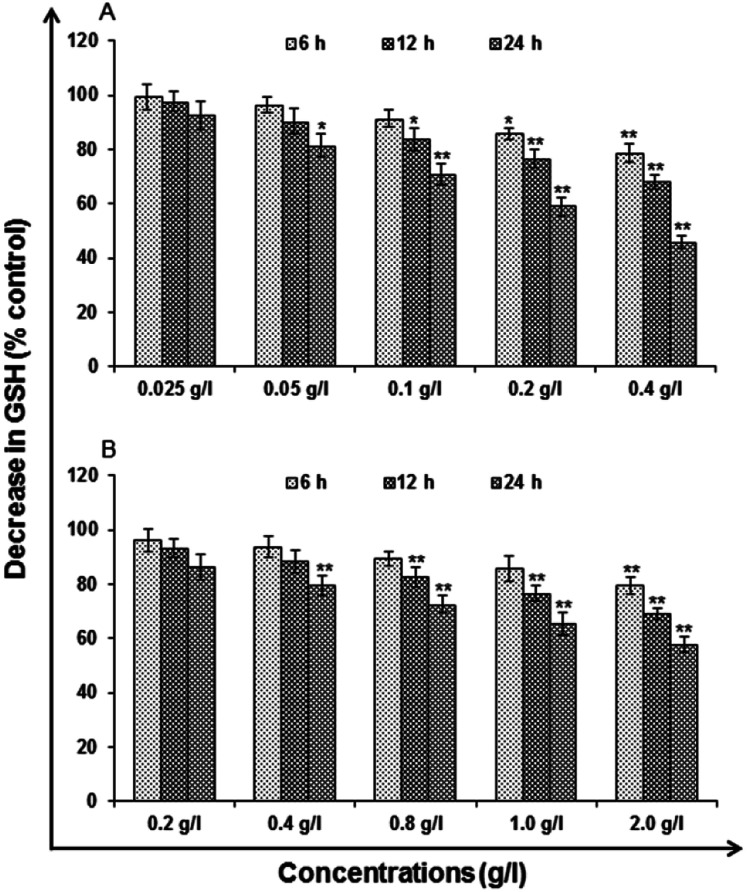
Change in levels of GSH activity in A549 cells following the exposure of (A) uncapped QDs and (B) capped QDs for 6, 12 and 24 h time periods assessed by micro plate reader. Data represented are mean ± SE of three identical experiments made in three replicate. *p < 0.05 = significant.

Cell exposed to uncapped QDs (0.2 and 0.4 g l^−1^) was found to reduce the levels of GSH significantly at every point of the time points 6 h (85.74 ± 2.00%, 78.68 ± 3.52%), 12 h (76.62 ± 3.23%, 67.94 ± 2.52%) and 24 h (58.98 ± 3.35%, 45.72 ± 2.23%) of control (Fig. 11A). However, the capped QDs (1.0 and 2.0 g l^−1^) show 6 h (85.78 ± 4.74%, 79.52 ± 3.24%), 12 h (76.64 ± 2.74%, 68.96 ± 2.09%) and 24 h (65.27 ± 4.20%, 57.63 ± 2.84%) of control (Fig. 11B). While lower concentration used could not pose such severe effects.

#### Lipid peroxidation (LPO)

**Fig. 12:**
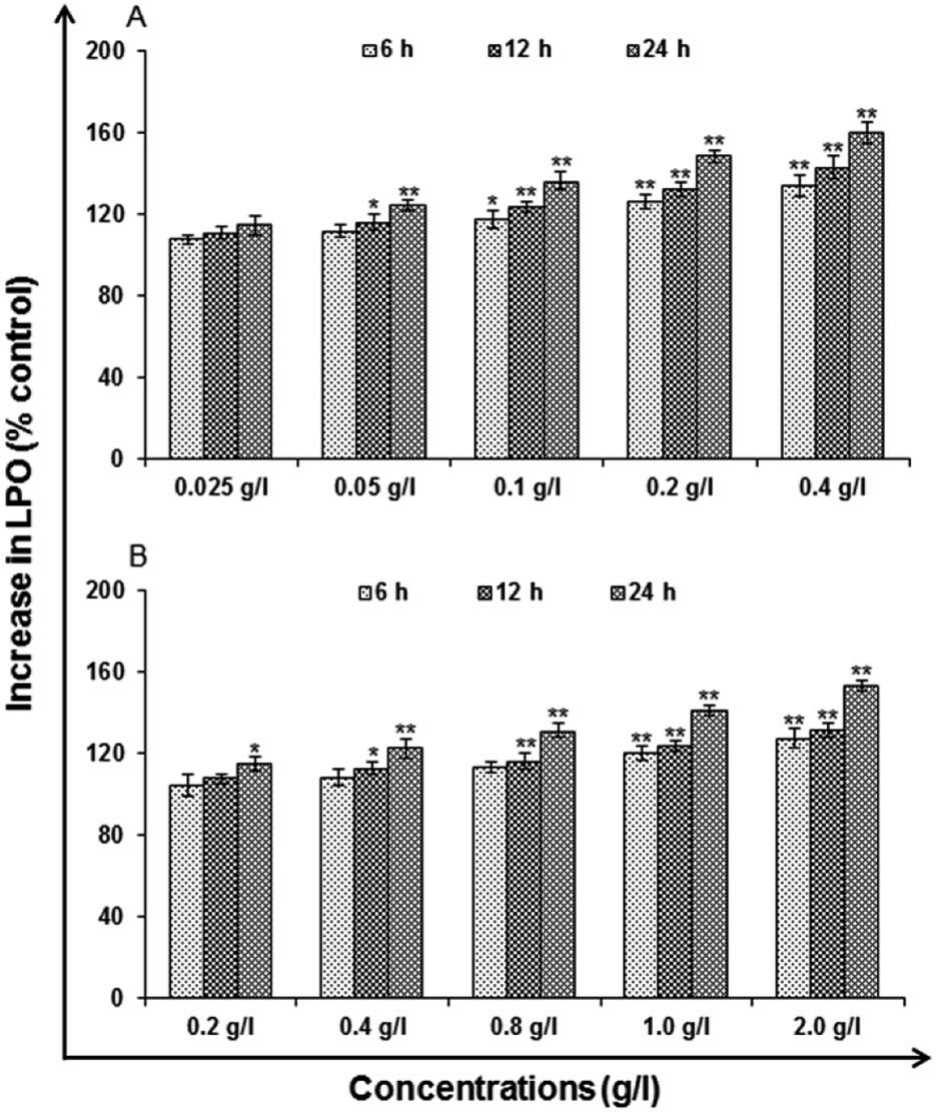
Change in levels of lipid peroxidation in A549 cells following the exposure of (A) uncapped QDs and (B) capped QDs for various time periods (6, 12 and 24 h) assessed by micro plate reader. Data represented are mean ± SE of three identical experiments made in three replicate. *p < 0.05 = significant.

Uncapped QDs at concentration 0.2 and 0.4 g l^−1^ induced significantly lipid per-oxidation at an exposure time of 6 h (126.34 ± 3.65%, 136.46 ± 5.33), 12 h (132.59 ± 3.56%, 143.39 ± 5.34%) and 24 h (148.76 ± 3.03%, 160.23 ± 5.32%) of control (Fig. 12A). In case of capped QDs at concentration 1.0 and 2.0 g l^−1^ significant induction of LPO was observed at 6 h (120.38 ± 3.24%, 127.63 ± 4.68), 12 h (123.64 ± 2.61%, 131.35 ± 3.63%) and 24 h (141.24 ± 2.76%, 153.31 ± 2.26%) of control (Fig. 12B). The stimulation of LPO was also found to vary with concentration and time.

#### Catalase (CAT) levels

**Fig. 13:**
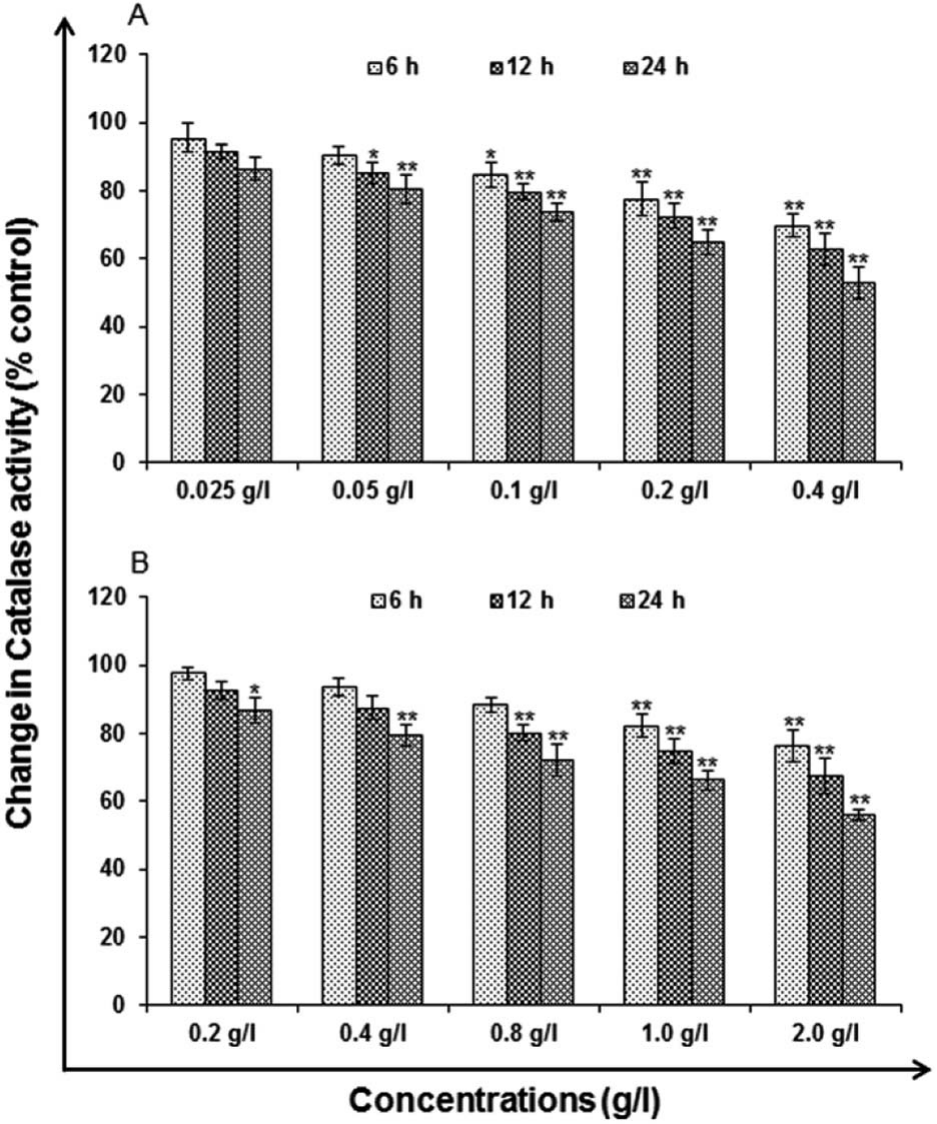
Change in levels of catalase activity in A549 cells following the exposure of (A) uncapped QDs and (B) capped QDs for various time periods (6, 12 and 24 h) assessed by micro plate reader. Data represented are mean ± SE of three identical experiments made in three replicate. *P < 0.05 = significant.

Major cut back in the action of catalase was observed at 0.2 and 0.4 g l^−1^, which was reported maximum at 24 h (64.92 ± 3.59%, 52.81 ± 4.69%) followed by 6 h (77.53 ± 4.80%, 69.74 ± 3.24%) and 12 h (72.46 ± 3.71%, 62.59 ± 4.69%) for uncapped QDs exposed cells (Fig. 13A). In case of capped QDs exposed cells, decrease in the activity of catalase was observed at 1.0 and 2.0 g l^−1^ and it also show maximum at 24 h (66.24 ± 2.75%, 55.82 ± 1.69%) followed by 6 h (82.36 ± 3.59%, 76.45 ± 4.68%) and 12 h (74.75 ± 3.58% and 67.62 ± 5.25) in comparison to unexposed control cells (Fig. 13B).

### Genotoxicity studies

#### Micronucleus assay

**Fig. 14:**
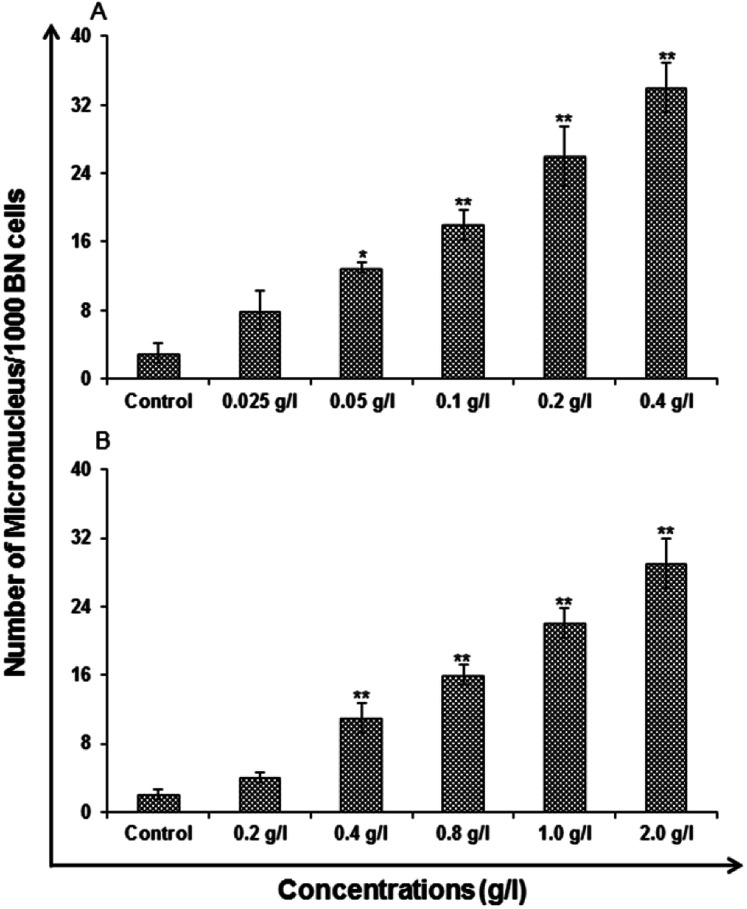
Genotoxicity/biosafety assessment of (A) uncapped QDs and (B) capped QDs in human lung epithelium cells-A549 (micronuclei assay). Cells were exposed to various concentrations for 24 h. Micronuclei were calculated by scoring a minimum of 1000 cells at 24 h. Values are given as mean ± standard error of the data obtained from three independent experiments and each experiment contained at least three replicates. *P < 0.05 = significant.

Micronucleus assay was conceded out to evaluate the accumulation of damage that is of genetic nature in the cells. The cells were developed in DMEM/F-12 medium. This condition was taken as control. The other set of cells exposed to different concentrations of uncapped and capped QDs for 24 h was measured as action group. It was observed that there is an increase in the MN frequency as compared to the cells grows in normal medium (Fig. 14). A significant change with increased induction of MN was found for the exposure of cells to 0.025, 0.05, 0.1, 0.2 and 0.4 g l^−1^ of uncapped QDs, *i.e.*, 8 ± 2.31, 13 ± 0.58, 18 ± 1.73, 26 ± 3.46 and 34 ± 2.89 MN/1000 cells at 24 h respectively (Fig. 14A). However, there was stimulation of micronucleus following the exposure of cells with 0.2, 0.4, 0.8, 1.0 and 2.0 g l^−1^ of capped QDs, *i.e.*, 4 ± 0.58, 11 ± 1.73, 16 ± 1.15, 22 ± 1.73 and 29 ± 2.89 MN/1000 cells at 24 h respectively (Fig. 14B).

#### Chromosomal aberration assay

**Fig. 15:**
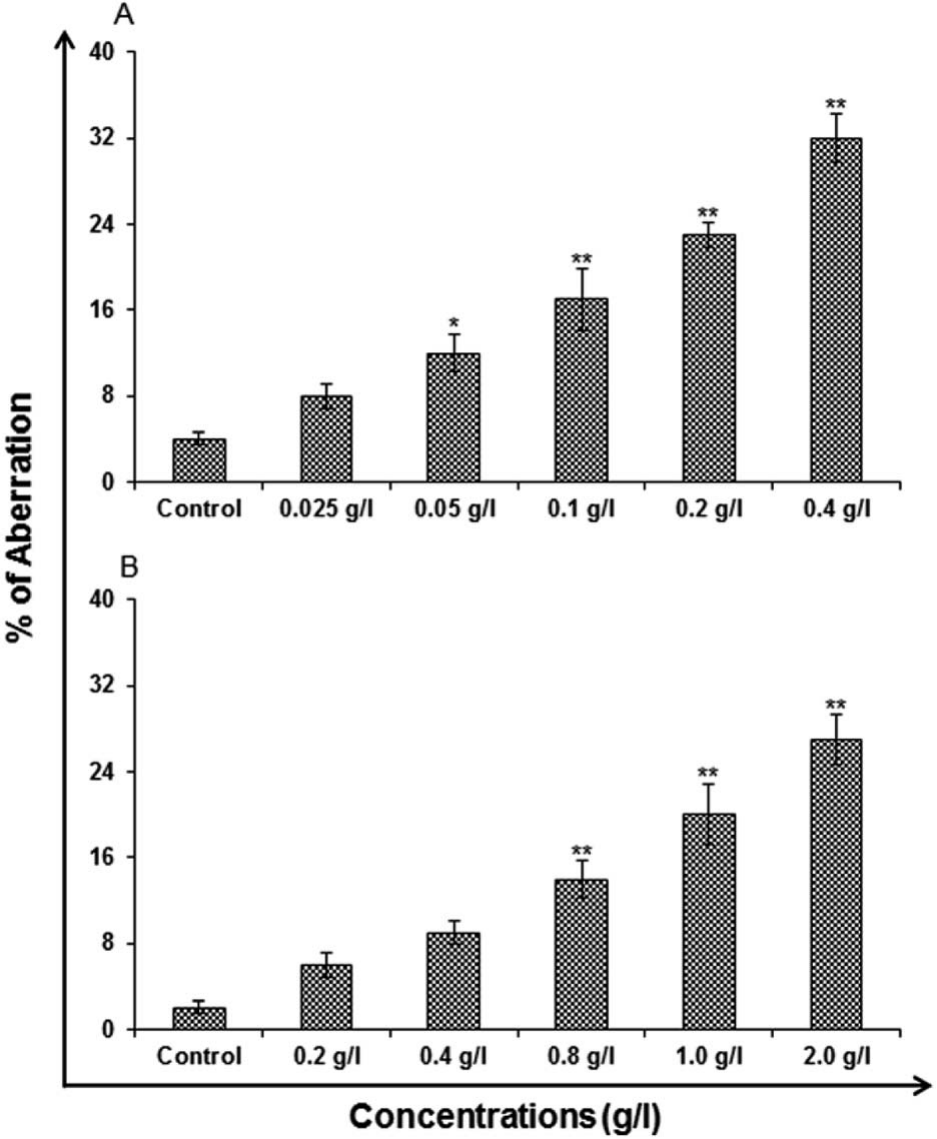
Genotoxicity/biosafety assessment of (A) uncapped QDs and (B) capped QDs in human lung epithelium cells-A549 (chromosomal aberration [CA]). Cells were exposed to various concentrations for 24 h. CA was scored at 24 h in the cells exposed to uncapped and capped QDs. Values are given as mean ± standard error of the data obtained from three independent experiments and each experiment contained at least three replicates. *P < 0.05 = significant.

The pattern was alike as that to MN assay. The most familiar aberrations were initiation of chromatid gaps and break type, followed by higher concentrations of uncapped and capped QDs. There were infrequent incidences of aneuploidy in the cells exposed to higher concentrations, *i.e.*, 0.2 and 0.4 g l^−1^ of uncapped and 1.0 and 2.0 g l^−1^ of capped QDs. The stimulation of CA was dose dependent, *i.e.*, 8 ± 1.15, 12 ± 1.73, 17 ± 2.89, 23 ± 1.15 and 32 ± 2.31 aberrations/100 in cells exposed to 0.025–0.4 g l^−1^ of uncapped QDs for 24 h respectively (Fig. 15A). The capped QDs show 6 ± 1.15, 9 ± 1.15, 14 ± 1.73, 20 ± 2.89 and 27 ± 2.31 aberrations/100 in cells that were exposed to 0.2–2.0 g l^−1^ for 24 h respectively (Fig. 15B).

**Fig. 16:**
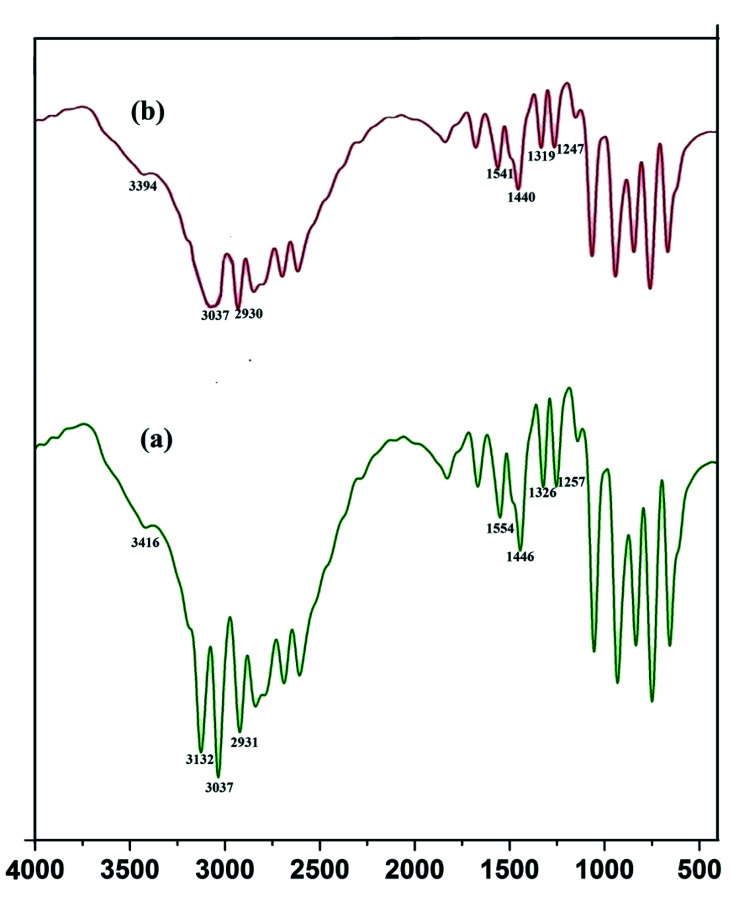
FTIR spectra of pure imidazole (a) and imidazole capped QDs (b).

## Conclusion

Mn doped ZnS QD is synthesized by employing aqueous route of synthesis. The imidazole is used as capping agent which does not alter the optical property or chemical structure of the synthesized material as is evident by the characterization using TEM, XRD and spectroscopy. Quantum chemical and experimental studies have been performed to understand the capping mechanism of ZnS quantum dot *via* imidazole. It is observed that most efficient capping is observed in the basic medium where deprotonation of the molecule leads to the formation of anionic species that forms a strong covalent bond with the zinc ion. The imidazole capped QD was further analysed and tested by toxicity parameters. Toxicity profiling of the synthesized QD was done at both cellular and genetic level. The toxicity study shows that uncapped QD shows toxic behavior as compared to imidazole capped QD.

## Conflicts of interest

This work does not involve any conflict of interest and all the authors have given their consent.

## Supplementary Material
